# Effectiveness, safety, and major adverse limb events in atrial fibrillation patients with concomitant diabetes mellitus treated with non-vitamin K antagonist oral anticoagulants

**DOI:** 10.1186/s12933-020-01043-2

**Published:** 2020-05-13

**Authors:** Yi-Hsin Chan, Hsin-Fu Lee, Pei-Ru Li, Jia-Rou Liu, Tze-Fan Chao, Lung-Sheng Wu, Shang-Hung Chang, Yung-Hsin Yeh, Chi-Tai Kuo, Lai-Chu See, Gregory Y. H. Lip

**Affiliations:** 1 The Cardiovascular Department, Chang Gung Memorial Hospital, Linkou, 33305 Taoyuan Taiwan; 2 College of Medicine, Chang Gung University, Taoyuan, 33302 Taiwan; 3 Microscopy Core Laboratory, Chang Gung Memorial Hospital, Linkou, 33305 Taoyuan Taiwan; 4 Graduate Institute of Clinical Medical Sciences, College of Medicine, Chang Gung University, Taoyuan, Taiwan; 5 New Taipei City Municipal Tucheng Hospital (Chang Gung Memorial Hospital, Tucheng branch, Taiwan), Taipei, Taiwan; 6 Department of Public Health, College of Medicine, Chang Gung University, No.259, Wenhua 1st Rd., Guishan Dist., Taoyuan City, 33302 Taiwan; 7 Division of Cardiology, Department of Medicine, Taipei Veterans General Hospital, Taipei, Taiwan; 8 Institute of Clinical Medicine, Cardiovascular Research Center, National Yang-Ming University, Taipei, Taiwan; 9 Center for Big Data Analytics and Statistics, Chang Gung Memorial Hospital, Taoyuan, Taiwan; 10 Biostatistics Core Laboratory, Molecular Medicine Research Center, Chang Gung University, Taoyuan, 33302 Taiwan; 11 Division of Rheumatology, Allergy and Immunology, Department of Internal Medicine, Chang Gung Memorial Hospital, Linkou, 33305 Taoyuan Taiwan; 12 Liverpool Centre for Cardiovascular Science, University of Liverpool and Liverpool Heart & Chest Hospital, Liverpool, UK

**Keywords:** Atrial fibrillation, Diabetes mellitus, Ischemic stroke, Major bleeding, NOACs, Warfarin

## Abstract

**Background:**

Evidence of adverse clinical outcomes for non-vitamin K antagonist oral anticoagulant (NOACs) and warfarin in patients with atrial fibrillation (AF) and diabetes mellitus are limited. We investigated the effectiveness, safety, and major adverse limb events for NOACs versus warfarin among diabetic AF patients.

**Methods:**

In this nationwide retrospective cohort study collected from Taiwan National Health Insurance Research Database, we identified a total of 20,967 and 5812 consecutive AF patients with diabetes taking NOACs and warfarin from June 1, 2012, to December 31, 2017, respectively. We used propensity-score stabilized weighting to balance covariates across study groups.

**Results:**

NOAC was associated with a lower risk of major adverse cardiovascular events (MACE) (adjusted hazard ratio (aHR):0.88; [95% confidential interval (CI) 0.78–0.99]; *P *= 0.0283), major adverse limb events (MALE) (aHR:0.72;[95% CI 0.57–0.92]; *P *= 0.0083), and major bleeding (aHR:0.67;[95% CI 0.59–0.76]; *P *< 0.0001) compared to warfarin. NOACs decreased MACE in patients of ≥ 75 but not in those aged < 75 years (*P* interaction = 0.01), and in patients with ischemic heart disease (IHD) compared to those without IHD (*P* interaction < 0.01). For major adverse limb events, the advantage of risk reduction for NOAC over warfarin persisted in high risk subgroups including age ≥ 75 years, chronic kidney disease, IHD, peripheral artery disease, or use of concomitant antiplatelet drugs.

**Conclusion:**

Among diabetic AF patients, NOACs were associated with a lower risk of thromboembolism, major bleeding, and major adverse limb events than warfarin. Thromboprophylaxis with NOACs should be considered in the diabetic AF population with a high atherosclerotic burden.

## Background

Atrial fibrillation (AF) is the most common cardiac arrhythmia globally, and is associated with a five-fold increased risk of stroke compared to patients without AF [1]. Diabetes mellitus (DM), insulin resistance, or obesity is an important risk factor of ischemic stroke and the development of new onset AF [2–4]. Around 40% AF patients have comorbid DM, and both are associated with a higher risk of ischemic stroke, acute coronary syndrome, and cardiovascular events [5].

The efficacy and safety of NAOCs have been studied in AF patients in association with several difficult treatment scenarios including the elderly, chronic kidney disease, valvular heart disease, or history of intracranial hemorrhage [6–8]. Current international guidelines recommend the use of non-vitamin K antagonist oral anticoagulants (NOACs) as effective, safer and more convenient alternatives to warfarin among patients with non-valvular AF, including those with DM [9, 10]. However, in post hoc analyses of the landmark NOACs trials, the safety profile regarding to the risk of major bleeding for dabigatran 110 mg or apixaban 5/2.5 mg over warfarin was diminished in AF patients comorbid with DM [11, 12]. Specifically, there were no clear benefits of NOACs over warfarin with regard to the risk of major bleeding in diabetic AF patients. In recent non-AF studies [13, 14], the value of NOACs in reducing major adverse limb events in patients at high vascular risk is also apparent.

In this study, we investigated the effectiveness, safety, and major adverse limb events for NOACs versus warfarin among diabetic AF patients, using a large population-based nationwide cohort study.

## Methods

We performed a retrospective nationwide cohort study using the Taiwan National Health Insurance Research Database (NHIRD), which contained health care information of more than 23 million Taiwan residents with a > 99% coverage rate of the entire population [15]. The NHIRD database contains each patient’s demographic data, outpatient clinic visits, hospitalizations, interventions and examinations, drug prescriptions, records of outpatient visits, and diagnosis of diseases. By using a consistent encrypting procedure, the original identification number of each patient in the NHIRD is encrypted and de-identified to protect patient privacy; therefore, informed consent was waived in the present study. This study was approved by the Institutional Review Board of the Chang Gung Medical Foundation (104-8079B and 201801427B0).

### Study design

**Fig. 1:**
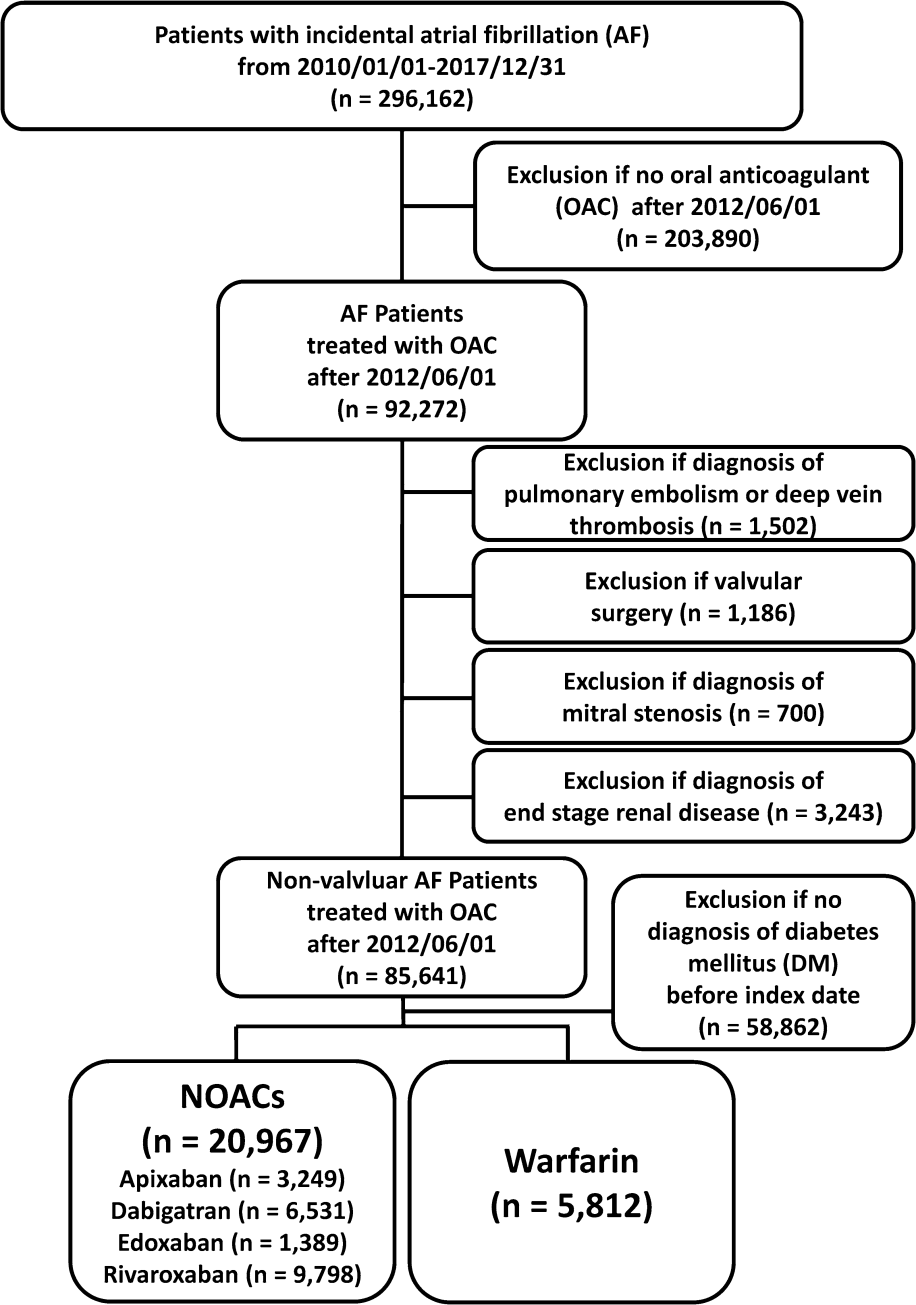
Enrollment of patients with concomitant non-vlavular atrial fibrillation (AF) and diabetes mellitus (DM). From June 1, 2012, to December 31, 2017, a total of 3249 (16%), 6531 (31%), 1389 (6%), and 9798 (47%) non-valvular AF patients comorbid with DM taking apixaban, dabigatran, edoxaban, and rivaroxaban and 5812 consecutive patients taking warfarin were enrolled in the present study. Abbreviations: *AF* atrial fibrillation, *DM* diabetes mellitus, *NOAC* non-vitamin K antagonist oral anticoagulant, *OAC* oral anticoagulant

The study identified a total of 296,162 patients diagnosed with AF using (*International Classification of Diseases (the ninth revision) Clinical Modification (ICD*-*9*-*CM)* codes (427.31) between January 1, 2010 and December 31, 2015 or using *ICD*-*10*-*CM codes* (I48)) between January 1, 2016 and December 31, 2018. A total of 92,272 AF patients treated with oral anticoagulants (OACs) after June 1, 2012 were identified. Patients who took more than one NOAC type during their treatment course were excluded from the present study. In order to establish a non-valvular AF cohort treated with OACs for stroke prevention, those patients with a diagnoses indicating deep vein thrombosis or pulmonary embolism, mitral stenosis, post valvular surgery, or joint replacement therapy within 6 months before the index date were excluded. Patients with end-stage renal disease were also excluded because NOACs are absolutely contraindicated in dialyzed patients in Taiwan. Finally, we included 85,641 non-valvular AF patients treated with OACs from June 1, 2012 to December 31, 2017. After excluding 58,864 non-valvular AF patients without a diagnosis of DM, a total of 20,967 and 5812 non-valvular AF patients comorbid with DM treated with NOACs and warfarin, respectively, were enrolled. The index date was defined as the first date of prescription for NOACs or warfarin. For those NOAC users with previous warfarin-exposure before (n = 6399), the index date was defined as the first date of prescription for their NOAC. The follow-up period was defined as the duration from the index date until the first occurrence of any study outcome independently, or until the end date of the study period (December 31, 2017), whichever came first. A flowchart of the study enrollment is shown in Fig. 1.

### Study outcomes

We reported several outcomes in the present study: (i) effectiveness outcomes: ischemic stroke/systemic embolism (IS/SE), acute myocardial infarction (AMI), and major adverse cardiac events (MACE) (defined as IS/SE or AMI); (ii) major lower limb outcomes: acute or chronic limb ischemia requiring revascularization procedures, lower limb amputation, and major adverse limb events (MALE) (defined as lower limb revascularization or amputation); (iii) safety outcomes: intracranial hemorrhage (ICH), major gastrointestinal bleeding, and all major bleeding events. All major bleeding events are defined as the summation of hospitalized events of ICH, major gastrointestinal bleeding, and other sites of critical bleeding. All study outcomes should be the primary discharge diagnosis to avoid misclassification. The diagnosis codes of NHIRD were shifted from ICD-9-CM to ICD-10-CM after January 1, 2016. The ICD-9-M and ICD-10-CM codes used to identify the baseline covariates and the study outcomes are summarized in Additional file 1: Table S1. The ICD-9-CM and ICD-10-CM codes used to identify the MALE outcomes are summarized in Additional file 1: Table S2 [16]. Patients may have had the same outcomes more than once during the study duration, but we only considered the same study outcome that occurred first.

### Covariates

Baseline covariates were obtained from any claim records with the diagnoses, medications, or procedures codes prior to the index date. A history of any prescription medicine was confined to medications taken at least once within 3 months before the index date. The definition of concomitant use of antiplatelet agent (APT) including aspirin, clopidogrel, ticlopidine, or ticagrelor was defined as APT duration > 3 months after drug index date. Bleeding history was confined to events within 6 months before the index date. The CHA_2_DS_2_-VASc score (congestive heart failure, hypertension, age 75 years or older for 2 points, DM, previous stroke or transient ischemic attack for 2 points, vascular disease, age 65 to 74 years, and female gender) was computed to predict the risk of thromboembolism in AF patients, [17]. The HAS-BLED score (hypertension, abnormal renal or liver function, stroke, bleeding history, labile INR, age 65 years or older, and APT or alcohol use) was computed to predict the risk of bleeding in AF patients treated with OACs [18].

### Statistical analysis

We used the method of propensity score stabilized weights (PSSWs) to balance the differences in baseline characteristics across the study groups [19]. The advantage of PSSWs is to provide an appropriate estimate of the main effect variance and to maintain the designated type I error by preserving the sample size of the original data. The PSSWs among study groups were obtained using the generalized boosted model (GBM), which can automatically determine the best functions of covariates, including interactions or polynomial terms, to obtain the optimal balance among study groups [20]. The advantage of PSSWs obtained by GBM is less affected by large weights [20]. All covariates in Table 1 except for CHA_2_DS_2_-VASc and HAS-BLED scores were included in the GBM, because CHA_2_DS_2_-VASc and HAS-BLED scores were already a combination of other covariates. The balance of potential confounders at baseline (index date) between each study group was assessed using the absolute standardized mean difference (ASMD) rather than statistical testing, because balance is a property of the sample and not of an underlying population. The value of ASMD ≤ 0.1 indicated an insignificant difference in potential confounders between the two study groups [21]. The incidence rates were computed using the total number of study outcomes during the follow-up period divided by person-years at risk. The risk of study outcomes for NOACs versus warfarin (reference) was obtained using survival analysis (Kaplan–Meier method and log-rank test for univariate analysis and Cox proportional hazards model for multivariate analysis). Subgroup analysis was performed to test whether the NOAC group continued to have a lower risk of clinical outcomes than the warfarin group in specific subgroup. It is noted that the PSSWs were re-estimated for each subgroup analysis so that the NOAC and warfarin subgroup maintained a balance of varied covariates across groups. Statistical significance was defined as a *P* value < 0.05. All statistical analyses were performed using SAS 9.4 (SAS Institute Inc., Cary, NC, USA).

**Table 1:** Baseline characteristics of non-valvular atrial fibrillation (AF) patients with diabetes mellitus (DM) before propensity score-based stabilized weights

	NOACs				NOACs (A + D+R + E) (n = 20,967)	Warfarin (n = 5812)	ASMD
	Apixaban n = 3249	Dabigatran n = 6531	Edoxaban n = 1389	Rivaroxaban n = 9798			NOACs
							Vs.
							Warfarin
Age							
(Mean ± STD)	76 ± 9.7	74 ± 9.7	75.4 ± 9.7	75.5 ± 9.7	75.1 ± 9.8	72.5 ± 11.6	0.2406
< 65	428 (13.2%)	1114 (17.1%)	196 (14.1%)	1345 (13.7%)	3083 (14.7%)	1664 (28.6%)	0.3470
65–74	976 (30%)	2229 (34.1%)	427 (30.7%)	3033 (31%)	6665 (31.8%)	1446 (24.9%)	
75–84	1210 (37.2%)	2354 (36%)	539 (38.8%)	3766 (38.4%)	7869 (37.5%)	1848 (31.8%)	
> 85	635 (19.5%)	834 (12.8%)	227 (16.3%)	1654 (16.9%)	3350 (16%)	854 (14.7%)	
Male	1704 (52.5%)	3800 (58.2%)	756 (54.4%)	5045 (51.5%)	11,305 (53.9%)	3080 (53%)	0.0185
CHA_2_DS_2_-VASc (mean ± STD)	4.7 ± 1.6	4.4 ± 1.6	4.5 ± 1.5	4.6 ± 1.6	4.5 ± 1.6	4.2 ± 1.8	0.1890
HAS-BLED (mean ± STD)	3.2 ± 1	3 ± 1	3.2 ± 1.1	3.1 ± 1	3.1 ± 1	2.9 ± 1.2	0.2299
Hypertension	2697 (83%)	5206 (79.7%)	1164 (83.8%)	7922 (80.9%)	16,989 (81%)	4446 (76.5%)	0.1109
Dyslipidemia	2200 (67.7%)	4102 (62.8%)	1011 (72.8%)	6425 (65.6%)	13,738 (65.5%)	3409 (58.7%)	0.1419
Chronic live disease	455 (14%)	834 (12.8%)	219 (15.8%)	1345 (13.7%)	2853 (13.6%)	779 (13.4%)	0.0060
Chronic kidney disease	880 (27.1%)	1164 (17.8%)	425 (30.6%)	2295 (23.4%)	4764 (22.7%)	1371 (23.6%)	0.0206
Chronic lung disease	413 (12.7%)	739 (11.3%)	165 (11.9%)	1389 (14.2%)	2706 (12.9%)	792 (13.6%)	0.0213
Gout	719 (22.1%)	1166 (17.9%)	328 (23.6%)	1994 (20.4%)	4207 (20.1%)	1113 (19.2%)	0.0230
Congestive heart failure	438 (13.5%)	684 (10.5%)	175 (12.6%)	1324 (13.5%)	2621 (12.5%)	919 (15.8%)	0.0951
Chronic ischemic heart disease	498 (15.3%)	764 (11.7%)	210 (15.1%)	1437 (14.7%)	2909 (13.9%)	844 (14.5%)	0.0186
Peripheral artery disease	296 (9.1%)	616 (9.4%)	132 (9.5%)	959 (9.8%)	2003 (9.6%)	544 (9.4%)	0.0066
Stroke	730 (22.5%)	1597 (24.5%)	205 (14.8%)	2259 (23.1%)	4791 (22.9%)	1034 (17.8%)	0.1260
Malignancy	370 (11.4%)	573 (8.8%)	165 (11.9%)	1059 (10.8%)	2167 (10.3%)	574 (9.9%)	0.0152
PCI	330 (10.2%)	417 (6.4%)	149 (10.7%)	868 (8.9%)	1764 (8.4%)	510 (8.8%)	0.0129
CABG	31 (1%)	25 (0.4%)	10 (0.7%)	71 (0.7%)	137 (0.7%)	90 (1.6%)	0.0859
History of bleeding	74 (2.3%)	117 (1.8%)	24 (1.7%)	222 (2.3%)	437 (2.1%)	148 (2.6%)	0.0307
Use of NSAIDs	918 (28.3%)	1551 (23.8%)	389 (28%)	2581 (26.3%)	5439 (25.9%)	1546 (26.6%)	0.0150
Use of PPI	451 (13.9%)	622 (9.5%)	174 (12.5%)	1187 (12.1%)	2434 (11.6%)	887 (15.3%)	0.1073
Use of H_2_ blocker	1132 (34.8%)	2068 (31.7%)	463 (33.3%)	3201 (32.7%)	6864 (32.7%)	2069 (35.6%)	0.0604
Use of ACEI/ARB	2205 (67.9%)	4470 (68.4%)	955 (68.8%)	6731 (68.7%)	14,361 (68.5%)	3984 (68.6%)	0.0012
Use of beta-blocker	2031 (62.5%)	3769 (57.7%)	925 (66.6%)	5939 (60.6%)	12,664 (60.4%)	3754 (64.6%)	0.0866
Use of verapamil or diltiazem	824 (25.4%)	1505 (23%)	260 (18.7%)	2551 (26%)	5140 (24.5%)	1666 (28.7%)	0.0940
Use of statin	1600 (49.3%)	2907 (44.5%)	701 (50.5%)	4574 (46.7%)	9782 (46.7%)	2263 (38.9%)	0.1564
Use of APT	480 (14.8%)	1383 (21.2%)	140 (10.1%)	1926 (19.7%)	3929 (18.7%)	2063 (35.5%)	0.3838
Use of metformin	1503 (46.3%)	3320 (50.8%)	584 (42%)	4488 (45.8%)	9895 (47.2%)	2567 (44.2%)	0.0608
Use of SU	1214 (37.4%)	2675 (41%)	454 (32.7%)	3574 (36.5%)	7917 (37.8%)	2312 (39.8%)	0.0415
Use of glinide	293 (9%)	498 (7.6%)	99 (7.1%)	846 (8.6%)	1736 (8.3%)	714 (12.3%)	0.1321
Use of acarbose	323 (9.9%)	654 (10%)	111 (8%)	924 (9.4%)	2012 (9.6%)	645 (11.1%)	0.0493
Use of glitazone	185 (5.7%)	381 (5.8%)	68 (4.9%)	463 (4.7%)	1097 (5.2%)	320 (5.5%)	0.0121
Use of insulin	927 (28.5%)	1388 (21.3%)	305 (22%)	2601 (26.6%)	5221 (24.9%)	2008 (34.6%)	0.2123
Use of SGLT2i	63 (1.9%)	54 (0.8%)	48 (3.5%)	106 (1.1%)	271 (1.3%)	20 (0.3%)	0.1054

*ACEI* angiotensin-converting-enzyme inhibitor, *AF* atrial fibrillation, *APT* antiplatelet agent, *ARB* angiotensin II receptor antagonists, *ASMD* absolute standardized mean difference, *CABG* coronary artery bypass grafting, *CHA*_*2*_*DS*_*2*_*-VASc* congestive heart failure, hypertension, age 75 years or older, diabetes mellitus, previous stroke/transient ischemic attack, vascular disease, age 65 to 74 years, female, *DM* diabetes mellitus, *HAS-BLED* hypertension, abnormal renal or liver function, stroke, bleeding history, labile INR, age 65 years or older, and antiplatelet drug or alcohol use, *INR* international normalized ratio, *NOAC* non-vitamin K antagonist oral anticoagulant, *NSAIDs* non-steroidal anti-inflammatory drugs, *PCI* Percutaneous coronary intervention, *PPI* proton pump inhibitor, *SGLT2i* sodium glucose co-transporters 2 inhibitor, *STD* standard deviation, *SU* Sulfonylurea

## Results

We identified a total of 20,967 and 5812 diabetic non-valvular AF patients treated with NOACs and warfarin, respectively. Among the NOAC group, there were 16%, 31%, 6%, and 47% patients taking apixaban, dabigatran, edoxaban, and rivaroxaban, respectively (Fig. 1). Among the NOAC group, there were 31% (n = 6399) patients who were warfarin-experienced before starting their NOAC (which was apixaban in 25% (n = 824), dabigatran in 34% (n = 2233), edoxaban in 23% (n = 325), and rivaroxaban in 31% (n = 3017)). Before PSSW, the NOAC group was older, and had a higher prevalence of hypertension, dyslipidemia, and stroke history than the warfarin group (ASMD > 0.1) (Table 1). The NOAC group also had a higher CHA_2_DS_2_-VASc and HAS-BLED score than the warfarin group before PSSW. After PSSW, both study groups were well-balanced in most characteristics (all ASMD < 0.1) (Table 2).

**Table 2:** Baseline characteristics of non-valvular AF patients with DM after propensity score-based stabilized weights

	NOACs				NOACs (A + D+R + E) (n = 20,967)	Warfarin (n = 5812)	ASMD
	Apixaban n = 3249	Dabigatran n = 6531	Edoxaban n = 1389	Rivaroxaban n = 9798			NOACs vs. Warfarin
Age							
(Mean ± STD)	75.6 ± 10	73.5 ± 10.1	74.9 ± 9.9	75 ± 10.1	74.6 ± 10.1	74.5 ± 10.3	0.0118
< 65	499.76 (15.6%)	1329.46 (20.3%)	226.27 (16.8%)	1619.19 (16.5%)	3674.69 (17.6%)	992.87 (17.8%)	0.0129
65–74	923.58 (28.8%)	2109.08 (32.3%)	396.11 (29.4%)	2894.27 (29.5%)	6323.04 (30.3%)	1658.22 (29.8%)	
75–84	1163.61 (36.3%)	2278.34 (34.9%)	506.75 (37.7%)	3656.43 (37.3%)	7605.13 (36.4%)	2030.02 (36.4%)	
> 85	620.66 (19.4%)	818.07 (12.5%)	216.63 (16.1%)	1627.86 (16.6%)	3283.22 (15.7%)	891.61 (16%)	
Male	1673.4 (52.2%)	3788.91 (58%)	730.05 (54.3%)	5027.21 (51.3%)	11,219.58 (53.7%)	2981.44 (53.5%)	0.0044
CHA_2_DS_2_-VASc (mean ± STD)	4.6 ± 1.6	4.3 ± 1.6	4.4 ± 1.5	4.5 ± 1.7	4.5 ± 1.6	4.4 ± 1.6	0.0184
HAS-BLED (mean ± STD)	3.2 ± 1.1	3 ± 1.1	3.1 ± 1.1	3.1 ± 1.1	3.1 ± 1.1	3 ± 1.1	0.0491
Hypertension	2646.3 (82.5%)	5147.03 (78.8%)	1117.08 (83%)	7823.89 (79.9%)	16,734.29 (80.1%)	4438.09 (79.6%)	0.0122
Dyslipidemia	2131.33 (66.5%)	4013.75 (61.4%)	964.54 (71.7%)	6272.89 (64%)	13,382.51 (64.1%)	3545.77 (63.6%)	0.0094
Chronic live disease	448.27 (14%)	836.45 (12.8%)	211.38 (15.7%)	1335.81 (13.6%)	2831.92 (13.6%)	745.06 (13.4%)	0.0056
Chronic kidney disease	884.71 (27.6%)	1165.76 (17.8%)	411.07 (30.6%)	2318.11 (23.7%)	4779.66 (22.9%)	1290.66 (23.2%)	0.0066
Chronic lung disease	415.03 (12.9%)	751.11 (11.5%)	161.41 (12%)	1403.16 (14.3%)	2730.7 (13.1%)	747.12 (13.4%)	0.0099
Gout	706.18 (22%)	1152.6 (17.6%)	317.42 (23.6%)	1975.72 (20.2%)	4151.94 (19.9%)	1105.89 (19.8%)	0.0009
Congestive heart failure	461.45 (14.4%)	721.17 (11%)	174.76 (13%)	1401.59 (14.3%)	2758.96 (13.2%)	770.92 (13.8%)	0.0185
Chronic ischemic heart disease	490.98 (15.3%)	764.79 (11.7%)	206.47 (15.3%)	1457.45 (14.9%)	2919.68 (14%)	740.87 (13.3%)	0.0202
Peripheral artery disease	294.56 (9.2%)	615.97 (9.4%)	126.94 (9.4%)	961.97 (9.8%)	1999.44 (9.6%)	531.89 (9.5%)	0.0010
Stroke	693.74 (21.6%)	1497.7 (22.9%)	189.25 (14.1%)	2161.77 (22.1%)	4542.45 (21.8%)	1175.9 (21.1%)	0.0160
Malignancy	365.73 (11.4%)	562.12 (8.6%)	160.26 (11.9%)	1055.43 (10.8%)	2143.54 (10.3%)	578.15 (10.4%)	0.0037
PCI	324.92 (10.1%)	414.45 (6.3%)	143.9 (10.7%)	889.57 (9.1%)	1772.84 (8.5%)	459.64 (8.3%)	0.0088
CABG	35.34 (1.1%)	30.41 (0.5%)	10.85 (0.8%)	90.42 (0.9%)	167.02 (0.8%)	44.51 (0.8%)	0.0001
History of bleeding	77.02 (2.4%)	118.84 (1.8%)	24.55 (1.8%)	229.26 (2.3%)	449.66 (2.2%)	119.51 (2.1%)	0.0006
Use of NSAIDs	908.1 (28.3%)	1567.68 (24%)	380.44 (28.3%)	2591.36 (26.5%)	5447.58 (26.1%)	1477.57 (26.5%)	0.0099
Use of PPI	470.21 (14.7%)	662.62 (10.1%)	177.39 (13.2%)	1261.57 (12.9%)	2571.79 (12.3%)	690.04 (12.4%)	0.0021
Use of H_2_ blocker	1136.31 (35.4%)	2108.48 (32.3%)	452.75 (33.6%)	3266.7 (33.3%)	6964.24 (33.3%)	1891.32 (33.9%)	0.0127
Use of ACEI/ARB	2179.74 (68%)	4473.09 (68.5%)	923.23 (68.6%)	6739.93 (68.8%)	14,315.99 (68.5%)	3807.33 (68.3%)	0.0048
Use of beta-blocker	2033.15 (63.4%)	3832.74 (58.7%)	905.14 (67.3%)	6041.78 (61.7%)	12,812.82 (61.4%)	3462.48 (62.1%)	0.0164
Use of verapamil or diltiazem	835.94 (26.1%)	1555.98 (23.8%)	260.29 (19.3%)	2647.44 (27%)	5299.65 (25.4%)	1421.74 (25.5%)	0.0032
Use of statin	1524.64 (47.5%)	2787.22 (42.7%)	662.45 (49.2%)	4428.17 (45.2%)	9402.48 (45%)	2467.58 (44.3%)	0.0150
Use of APT	557.85 (17.4%)	1634.37 (25%)	163.4 (12.1%)	2288.62 (23.4%)	4644.24 (22.2%)	1275.11 (22.9%)	0.0156
Use of metformin	1464.25 (45.7%)	3276.26 (50.1%)	551.96 (41%)	4424.87 (45.2%)	9717.34 (46.5%)	2605.79 (46.8%)	0.0047
Use of SU	1213.44 (37.8%)	2703.68 (41.4%)	443.81 (33%)	3613.42 (36.9%)	7974.35 (38.2%)	2150.56 (38.6%)	0.0085
Use of glinide	316.99 (9.9%)	545.52 (8.4%)	104.16 (7.7%)	941.11 (9.6%)	1907.78 (9.1%)	524.66 (9.4%)	0.0098
Use of acarbose	327.99 (10.2%)	675.87 (10.3%)	110.92 (8.2%)	954.94 (9.8%)	2069.72 (9.9%)	564.11 (10.1%)	0.0072
Use of glitazone	187.7 (5.9%)	380.2 (5.8%)	64.64 (4.8%)	470.95 (4.8%)	1103.49 (5.3%)	294.35 (5.3%)	0.0001
Use of insulin	981.92 (30.6%)	1499.29 (22.9%)	316.6 (23.5%)	2823.21 (28.8%)	5621.01 (26.9%)	1541.1 (27.7%)	0.0168
Use of SGLT2i	62.44 (2%)	52.38 (0.8%)	48.25 (3.6%)	109.87 (1.1%)	272.94 (1.3%)	19.75 (0.4%)	0.1057

The abbreviations as in Table 1

**Fig. 2:**
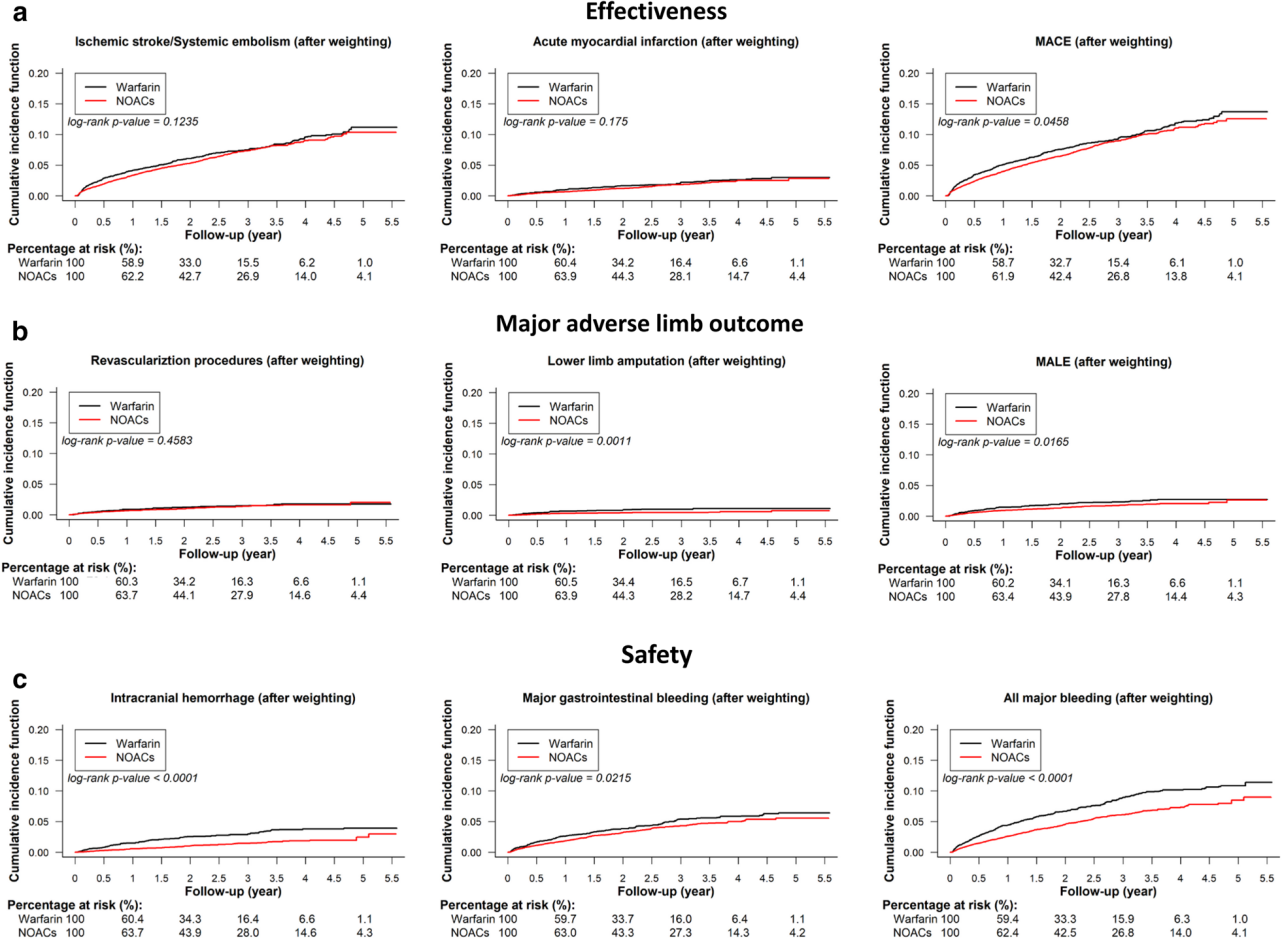
Cumulative incidence curves of outcomes for diabetic AF patients taking oral anticoagulants after propensity score stabilized weighting (PSSW). Cumulative incidence curves of effectiveness outcomes including ischemic stroke/systemic embolism (IS/SE), acute myocardial infarction (AMI), and major adverse cardiovascular events (MACE) **a**, major adverse limb events including lower extremity revascualization procedure, lower limb amputation, and major adverse limb events (MALE) **b,** and safety outcomes including intracranaial hemorrhage (ICH), major gastrointestinal bleeding, and all major bleeding **c** for AF patients with concomiant DM taking oral anticoagulants after PSSW are presented. NOAC was associated with a lower risk of MACE, MALE, and all major bleeding events than warfarin among AF patients with concomitant DM. Abbreviations: *AF* atrial fibrillation, *AMI* acute myocardial infarction, *DM* diabetes mellitus, *ICH* intracranial hemorrhage, *IS/SE* ischemic stroke/systemic embolism, *MACE* major adverse cardiovascular events, *MALE* major adverse limb events, *NOAC* non-vitamin K antagonist oral anticoagulants, *PSSW* propensity score stabilized weighting

For the effectiveness outcome, the DOAC group had a lower cumulative risk of MACE when compared to the warfarin group after PSSW. For the major adverse limb events, DOAC was associated with a lower cumulative risk of lower limb amputation and MALE than warfarin. For the safety outcomes, DOAC use was associated with a lower cumulative risk of ICH, major gastrointestinal bleeding, and all major bleeding than warfarin (Fig. 2). Before PSSW, NOACs were associated with a lower risk of all study outcomes than warfarin (Additional file 2: Figure S1).

**Fig. 3:**
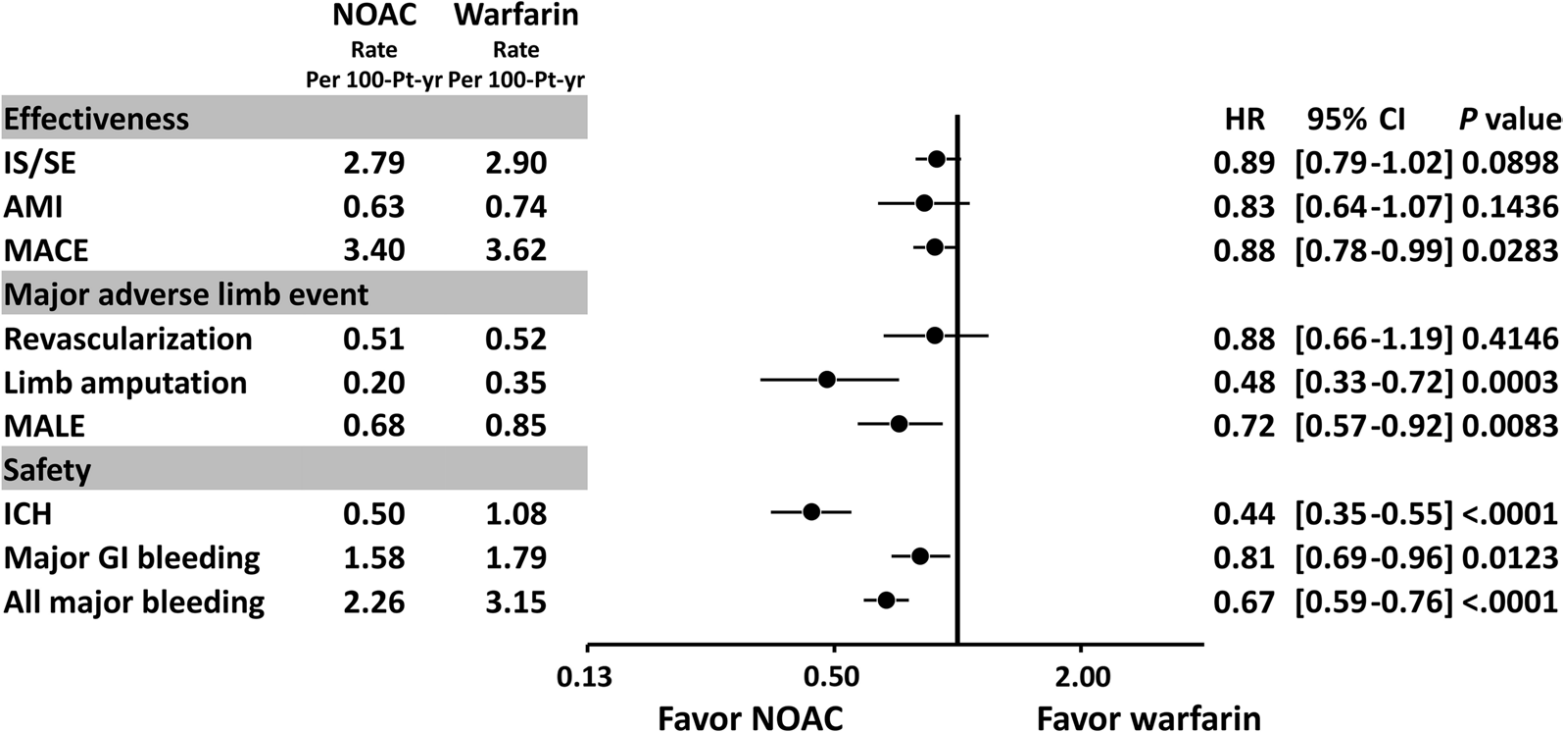
Forest plot of hazard ratio (HR) of effectiveness, major lower limb outcomes, and safety outcomes for NOACs vs. warfarin among non-valvular AF patients comorbid with DM after PSSW. NOAC was associated with a lower risk of MACE, major adverse limb events (MALE), and major bleeding than warfarin among non-valvular AF patients with concomitant DM. Abbreviations: *AF* atrial fibrillation, *AMI* acute myocardial infarction, *CI* confidential interval, *GI* gastrointestinal, *HR* hazard ratio, *ICH* intracranial hemorrhage, *IS/SE* ischemic stroke/systemic embolism, *MACE* major adverse cardiovascular events, *MALE* major adverse limb events, *NOAC* non-vitamin K antagonist oral anticoagulants, *PSSW* propensity score stabilized weighting

For the effectiveness outcome, the NOAC group had a lower risk of MACE (hazard ratio (HR): 0.88; 95% confidential interval (CI) [0.78–0.99]; *P* = 0.0283) when compared to the warfarin group after PSSW. For the major lower limb outcomes, DOAC was associated with a lower risk of lower limb amputation (HR: 0.48; 95% CI [0.33–0.72]; *P* = 0.0003) and MALE (HR: 0.72; 95% CI [0.57–0.92]; *P* = 0.0083) than warfarin. For the safety outcomes, NOAC was associated with a lower risk of ICH (HR: 0.44; 95% CI [0.35–0.55]; *P* < 0.0001), major gastrointestinal bleeding (HR: 0.81; 95% CI [0.69–0.96]; *P* = 0.0123), and all major bleeding (HR: 0.67; 95% CI [0.59–0.76]; *P* < 0.0001) than warfarin (Fig. 3).

### Sensitivity test

Sensitivity analyses were performed by using a multivariate Cox proportional-hazards model, rather than the PSSW, to test if the results were still consistent with the main analysis by using PSSW. The model was adjusted for all baseline characteristics listed in Table 1 except for CHA_2_DS_2_-VASc and HAS-BLED scores. Consistent with the main analysis by using PSSW, the use of NOAC was associated with a lower risk of MACE (HR: 0.88; 95% CI [0.78–0.99]; *P* = 0.0287), MALE (HR: 0.73; 95% CI [0.58–0.92]; *P* = 0.0099), and major bleeding (HR: 0.70; 95% CI [0.62–0.80]; *P* < 0.0001) compared to warfarin, after multivariate adjustment (Additional file 3: Figure S2).

### Subgroup analysis of different NOACs versus warfarin

**Fig. 4:**
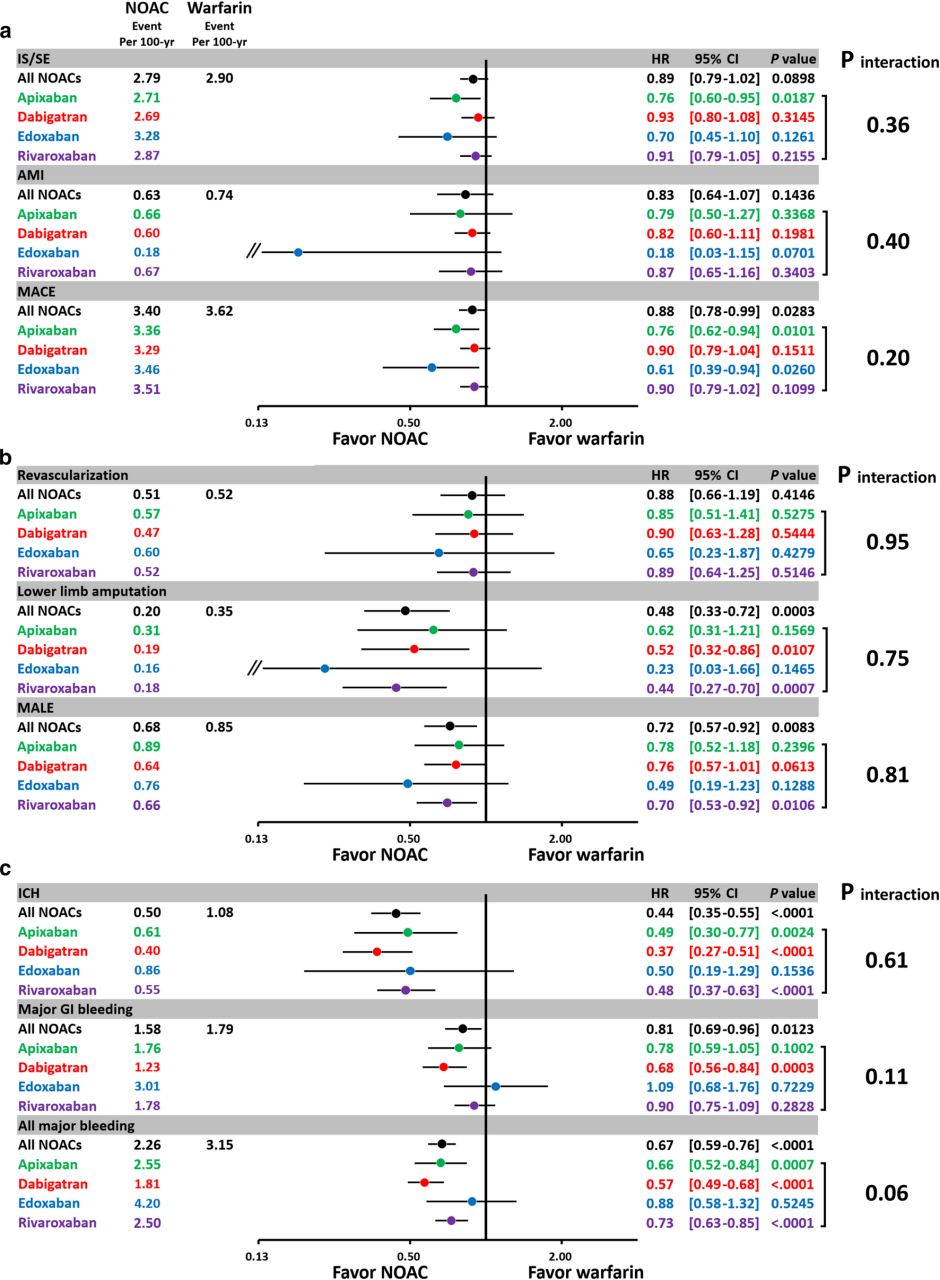
Forest plot of HR of effectiveness, major lower limb outcomes, and safety outcomes for each NOAC vs. warfarin among non-valvular AF patients with concomitant DM taking oral anticoagulants after PSSW. In general, the advantage of effectiveness, major adverse limb outcome, and safety for NOAC over warfarin persisted in four NOACs (*P* interaction all > 0.05). The abbreviations as in Fig. 3

Subgroup analysis was performed to determine whether different NOACs were superior to warfarin regarding to the effectiveness, major adverse limb events, and safety among subgroups. There were 66%, 89%, 68%, and 95% patients taking low-dose apixaban (2.5 mg twice daily), dabigatran (110 mg twice daily), edoxaban (30 mg daily), and rivaroxaban (15/10 mg daily) among the NOAC group, respectively. In general, the advantage of effectiveness, major limb outcome, and safety for NOAC over warfarin persisted in four NOAC subgroup (*P* interaction all > 0.05) (Fig. 4).

### Subgroup analysis

**Fig. 5:**
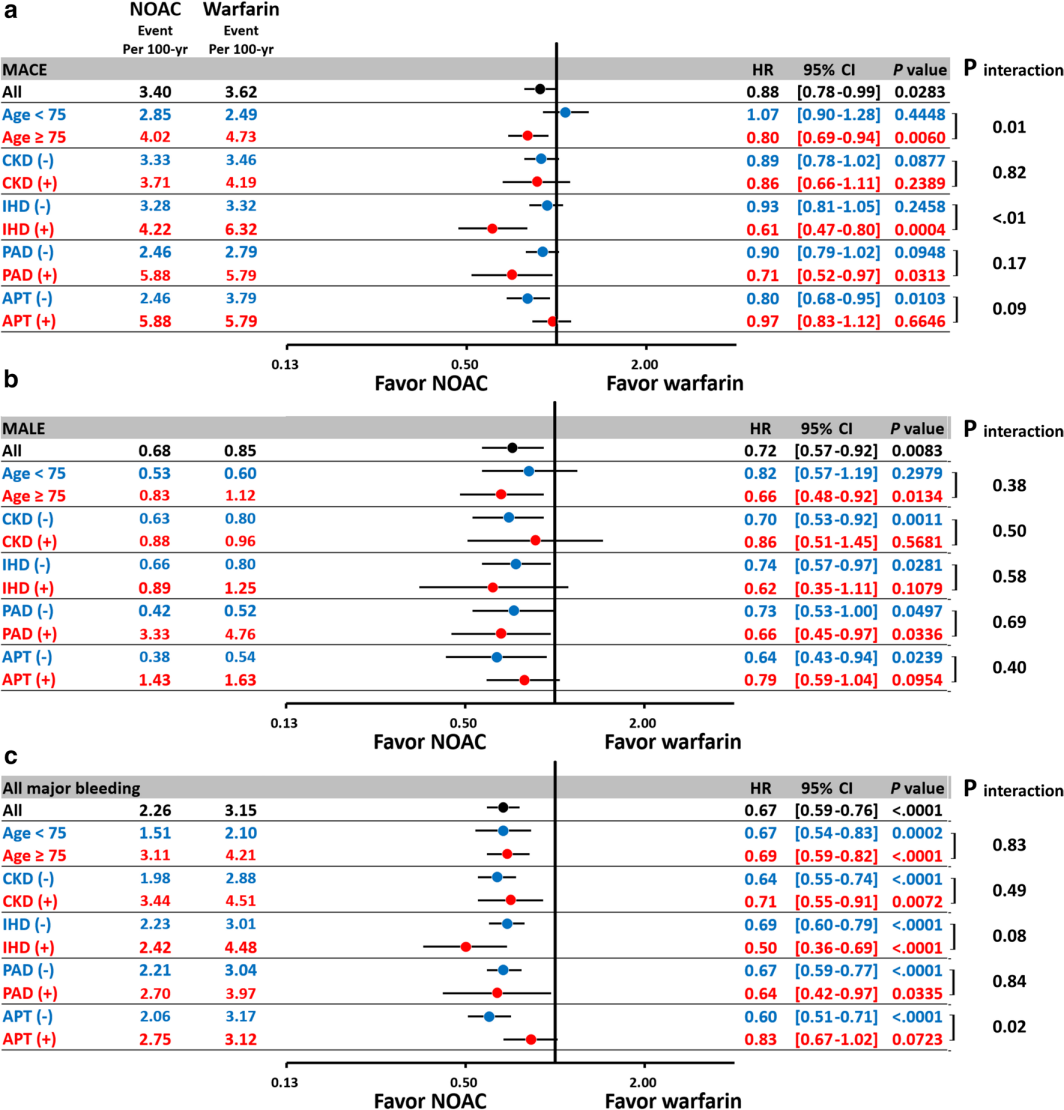
Subgroup analysis of forest plot of HR for NOAC vs. warfarin among non-valvular AF patients with concomitant DM after PSSW. In general, the subgroup analysis showed consistent results for MACE, MALE, and major bleeding for NOACs vs. warfarin among those patients with ≥ 75 years of age, the presence of chronic kidney disease (CKD), ischemic heart disease (IHD), peripheral artery disease (PAD), and use of concomitant antiplatelet agent (APT) as the main analysis. Furthermore, NOAC reduced MACE more in diabetic AF patients with a high atherosclerotic burden including elderly, the presence of IHD or PAD. Abbreviations: *APT* antiplatelet agent, *CKD* chronic kidney disease, *IHD* ischemic heart disease, *PAD* peripheral artery disease. Other abbreviations as in Fig. 3

In general, the subgroup analysis showed consistent results for MACE, MALE, and all major bleeding for NOACs versus warfarin among those patients with ≥ 75 years of age, the presence of chronic kidney disease (CKD), ischemic heart disease (IHD), peripheral artery disease (PAD), and use of concomitant APT consistent with the main analysis (Fig. 5). NOACs decreased the risk of MACE in patients aged ≥ 75 years of age but not in patients of < 75 years of age (*P* interaction = 0.01) and in patients with concomitant IHD than in those without concomitant IHD (*P* interaction < 0.01). For the subgroup analysis of patients taking concomitant APT, there was a lower risk of major bleeding for NOAC versus warfarin especially in the APT (-) subgroup (*P* interaction = 0.02).

## Discussion

To our best knowledge, this is the largest population-based study to investigate the effectiveness, safety, and major limb outcomes for the four NOACs vs. warfarin in Asian population comorbid with AF and DM. Our results indicated that NOACs were associated with a lower risk of MACE, MALE, and all major bleeding when compared to warfarin among AF patients comorbid with DM. Second, the advantage of effectiveness, major limb outcome, and safety for NOAC over warfarin persisted in four NOAC subgroups (*P* interaction all > 0.05) and in high risk subgroups. Third, NOAC reduced MACE more in diabetic AF patients with a high atherosclerotic burden including the elderly, and the presence of IHD or PAD.

### Comparisons of four NOACs vs. warfarin in diabetic AF population

The meta-analysis of the four landmark NOAC trials indicated that NOACs significantly reduced the composite efficacy endpoint when compared to warfarin both in non-valvular AF patients with diabetes and in those without diabetes, with no significant interaction by diabetes status and treatment [22–24]. In a post hoc analysis of ARISTOTLE trial, a significant interaction was noted between diabetes status and treatment regarding the risk of major bleeding (*P* interaction = 0.0034), suggesting that apixaban reduces more major bleeding than warfarin only among AF patients without diabetes [12]. A post hoc analysis of the RE-LY trial showed a comparable risk of major bleeding in AF patients with diabetes treated with dabigatran 110 mg twice daily vs. warfarin (HR: 0.91; 95% CI [0.70–1.19]), which was in contrast to a significantly lower risk of major bleeding (HR: 0.76; 95% CI [0.64–0.90]) in non-diabetic patients treated with dabigatran 110 mg twice daily vs. warfarin [11]. In the post hoc analysis of ROCKET-AF study, rivaroxaban showed a comparable risk of major bleeding to warfarin either in the diabetic or non-diabetic subgroup, and there was no significant interaction between diabetic status and the risk of bleeding [25]. Finally, in the post hoc analysis of ENGAGE-AF TIMI 48 trial, edoxaban had a significantly lower risk of major bleeding than warfarin both in the diabetic (HR: 0.78; 95% CI [0.63–0.95]) and non-diabetic subgroups (HR: 0.81; 95% CI [0.69–0.95]) (*P* interaction > 0.10) [23, 24]. In summary, the advantage of NOACs over warfarin in efficacy generally persisted in diabetic subgroup treated with four NAOCs, whereas the advantage of safety profiles regarding to the risk of major bleeding for NOACs over warfarin had some conflicting results in diabetic AF population treated with NOACs, especially in case of apixaban and dabigatran 110 mg.

The present study indicated that NOACs was associated with a significantly lower MACE than warfarin in those diabetic AF population with a high atherosclerotic burden like the presence of concomitant IHD or PAD (Fig. 4). For AF patient comorbid with IHD or PAD, guidelines recommend the use of oral anticoagulant (OAC) rather than APT [9, 10]. However, there are no data or guideline recommendations specifically focused on the optimal treatment for diabetic AF patients with concomitant IHD or PAD [10, 26, 27]. Previous studies have indicated that warfarin may increase vascular calcification and osteoporotic bone fracture via inhibition of the activation of matrix and bone G1a protein, and may increase coronary or peripheral vascular calcification, thus potentially influencing symptoms and outcomes in patients with IHD or PAD [28–33]. Furthermore, patients with IHD or PAD have a higher risk of bleeding events compared to those without IHD or PAD, and the bleeding events may further increase the risk of ischemic events in the IHD or PAD, for example, discontinuation of OAC due to bleeding may cause consequent ischemic event like AMI or critical limb ischemia [34]. Our present study demonstrates the benefit of NOACs over warfarin regarding to the effectiveness and safety outcomes even in a very high risk patient population comorbid with AF, diabetes, and IHD/PAD.

### Major limb outcomes for NOACs vs. warfarin in diabetic AF population

Data regarding to the major adverse limb events for AF patients treated with NOAC vs. warfarin are limited. We are only aware of one retrospective study investigating the major limb outcomes for diabetic AF patients treated with NOAC vs. warfarin [35]. Baker et al. studied 10,700 and 13,946 diabetic AF patients treated with rivaroxaban and warfarin, respectively, by using a claims database in USA, whereby rivaroxaban was associated with a 25% reduced risk of MACE and a 63% reduced risk of MALE compared to warfarin, with no difference in major bleeding [35]. Our present study shows that NOACs, with nearly 50% of whom treated with rivaroxaban, were also associated with a significantly lower risk of MALE than warfarin in diabetic AF patients. Although there is no difference for the advantage on MALE for different NOACs over warfarin (*P* interaction = 0.81 within four NOACs), the other three NOACs except for rivaroxaban showed non-significantly lower risk of MALE than warfarin among the diabetic patients, possibly due to a smaller sample size of other three NOACs when compared to rivaroxaban (Fig. 3).

Recently, the COMPASS trial showed a strategy of combined therapy with aspirin and rivaroxaban (2.5 mg twice per day) or rivaroxaban alone (5 mg twice per day) was associated with a significantly lower risk of major adverse limb events than aspirin alone in ~ 27,000 patients with stable atherosclerotic vascular disease, nearly 45% of whom had comorbid diabetes [14]. Although the COMPASS trial differ from our present study in that it did not enroll AF patients, used a lower dose of rivaroxaban, and used aspirin but not warfarin as a comparator, the COMPASS trial firstly demonstrated that anticoagulant regimen with rivaroxaban indeed provide an extra benefit in improving MALE outcome in patients with a high atherosclerotic burden when compared to the current standard treatment. Until now, there are no large randomized controlled studies evaluating the major limb outcome for AF patients with a high atherosclerotic burden or AF treated with other three NOACs.

### Limitations

The present study has several limitations. First, our study is a retrospective cohort study. Although the use of inverse propensity score weighting with adjustment of several variables allowed the balance of baseline comorbidities among the study groups, selection bias and residual confounding by unobserved or unmeasured variables could not be excluded in the present study. Second, misclassification and miscoding of the baseline comorbidities and study outcomes is a potential limitation. Third, laboratory data such as international normalized ratio (INR) for patients treated with warfarin were not obtained in the NHIRD database; indeed, Asian populations treated with warfarin generally have a much lower time in therapeutic range (TTR) for an INR target 2.0 to 3.0 compared to other regions of the world [36, 37]. Hence, the superiority of NOACs over warfarin may be partly due to low TTR for those patients treated with warfarin in the present study [38]. Fourth, four NOACs and warfarin prescribed had varying rates of renal excretion and, thus, decisions regarding the use of a specific NOAC or warfarin would have been guided by the renal function of each patient (e.g., a perceived risk may result in conscious avoidance in use of NOACs in specific patient populations). Renal function lab data for each patient are lacking in the NHIRD, and ICD coding indicating an impaired renal function was dependent on each physician’s choice. Therefore, residual confounding factors including the renal function across the exposure groups could not be excluded. In addition, there was a high prevalence of low-dose NOAC prescriptions in the present AF cohort. The lack of both body weight and renal function data makes us difficult to determine if those AF patients treated with low-dose NOACs were correctly prescribed with an “adjusted” or “off-label” low-dose NOACs. Fifth, glycated hemoglobin is directly associated with risk of stroke in diabetic patients with AF [39]; however, we are unable to determine the quality of glycemic control for each diabetic AF patient due to lack of glycated hemoglobin data. Finally, for the issue of MALE outcome, we were unable to differentiate whether the outcome of lower limb amputation or revascularization was due to a pure cardio-embolic event caused by AF itself or an atherothrombotic event caused by DM related atherosclerosis and PAD. Diabetic patients have a higher prevalence of PAD than other populations, and the underlying pathology for PAD related critical limb ischemia is mainly mediated by atherothrombotic events [40]. Nevertheless, the COMPASS trial has demonstrated that use of a NOAC was beneficial in reducing atherothrombotic events in PAD patients, whereby nearly 45% had comorbid diabetes [14].

## Conclusions

Among diabetic AF patients, NOACs were associated with a lower risk of thromboembolism, major bleeding, and major adverse limb events than warfarin. Thromboprophylaxis with NOACs should be considered in the diabetic AF population with a high atherosclerotic burden.

## Supplementary information

**Additional file 1: Table S1.** International Classification of Disease (9thand 10thedition) Clinical Modification (ICD 9-CMand ICD 10-CM) codes used to define the co-morbidities and clinical outcome in the study cohort. **Table S2.** International Classification of Disease (9thand 10thedition) Clinical Modification (ICD 9-CM and ICD 10-CM) codes used to define the major adverse limb outcome in the study cohort.

**Additional file 2: Figure S1.** Cumulative incidence curves of outcomes for atrial fibrillation (AF) patients with concomitant diabetes mellitus (DM) taking oral anticoagulants before propensity score stabilized weighting (PSSW).

**Additional file 3: Figure S2.** Forest plot of hazard ratio (HR) of effectiveness, major lower limb outcomes, and safety outcomes for NOACs vs. warfarin among non-valvular AF patients comorbid with DM, after multi-variate adjustment.

## Data Availability

The datasets used in this study were only available in the Applied Health Research Data Integration Service from National Health Insurance Administration, Taiwan. The SAS programs (codes) involved for this study are available from the corresponding author on reasonable request.

## Abbreviations

AF: Atrial fibrillation
AMI: Acute myocardial infarction
APT: Antiplatelet agent
CHA2DS2-VASc: Congestive heart failure, hypertension, age 75 years or older, diabetes mellitus, previous stroke/transient ischemic attack, vascular disease, age 65 to 74 years, female
CKD: Chronic kidney disease
DM: Diabetes mellitus
HAS-BLED: Hypertension, abnormal renal or liver function, stroke, bleeding history, labile INR, age 65 years or older, and antiplatelet drug or alcohol use
ICH: Intracranial hemorrhage
IHD: Ischemic heart disease
IS/SE: Ischemic stroke/systemic embolism
MACE: Major adverse cardiovascular event
MALE: Major adverse limb event
NOAC: Non-vitamin K antagonist oral anticoagulants
PAD: Peripheral artery disease
PSSW: Propensity score stabilized weighting
