# Supplementary material for: Effectiveness, safety, and major adverse limb events in atrial fibrillation patients with concomitant diabetes mellitus treated with non-vitamin K antagonist oral anticoagulants
Source: Cardiovasc Diabetol. 2020 May 13;19:63. doi: 10.1186/s12933-020-01043-2 (PMC7222472; doi:10.1186/s12933-020-01043-2)
Supplement: Supplementary file 1 — Additional file 1: Table S1. International Classification of Disease (9thand 10thedition) Clinical Modification (ICD 9-CMand ICD 10-CM) codes used to define the co-morbidities and clinical outcome in the study cohort. Table S2. International Classification of Disease (9thand 10thedition) Clinical Modification (ICD 9-CM and ICD 10-CM) codes used to define the major adverse limb outcome in the study cohort. [file 12933_2020_1043_MOESM1_ESM.doc]

**ADDITIONAL FILE**

**Table S1**

International Classification of Disease (9^th^ and 10^th^ edition) Clinical Modification (ICD 9-CM and ICD 10-CM) codes used to define the co-morbidities and clinical outcome in the study cohort

| Disease | ICD-9 Codes | ICD-10 Codes | Diagnosis definition |
| --- | --- | --- | --- |
| Atrial fibrillation | 427.31 | I48 | Discharge or outpatient department ≥2 |
| Ischemic stroke | 433, 434, 436 | I63, I64 | Discharge |
| Systemic embolism | 444 | I74 | Discharge |
| [Transient ischemic attack](http://en.wikipedia.org/wiki/Transient_ischemic_attack) | 435 | G45 | Discharge |
| Acute Myocardial infarction | 410 | I21-I23 | Discharge |
| Peripheral arterial disease | 440.0, 440.2, 440.3, 440.8, 440.9, 443,  444.0, 444.22, 444.8, 444.9, 447.9, 440.0, 38.08, 38.18, 38.38, 38.48, 38.68, 38.88,  39.50, 39.7, 39.90, 39.25, 39.26, 39.29  84.10-84.15,  84.16-84.19 | I70.0, I70.2, I70.9, I70.3, I70.8, I75.89, I70.9, I73.0, I73.1, I73.8, I73.9, I79.1, I79.8, I74.01, I74.09, I74.3, I74.4, I74.5, I74.8, I74.9, I77.9, I70.0, 041,045,047,049,04B,04C,04H, 04J,04L,04N,04P,04Q,04R,04S, 04U,04V,04W  Location:(C,D,E,F,H,J,K,L,M,N,P,Q,R,S,T,U,V,W,Y)  0Y67, 0Y68, 0Y6C, 0Y6D, 0Y6F, 0Y6G, 0Y6H, 0Y6J, 0Y6M, 0Y6N, 0Y6P, 0Y6Q, 0Y6R, 0Y6S, 0Y6T, 0Y6U, 0Y6V, 0Y6W, 0Y6X, 0Y6Y | Discharge or Outpatient department ≥2 |
| Ischemic heart disease | 410, 411, 412, 413, 414 | I21-I25 | Outpatient department ≥2 |
| Congestive heart failure | 428 | I11.0, I13.0, I13.2, I42.0, I50, I50.1, I50.9 | Discharge |
| Hypertension | 401, 402 | I10-I16 | Outpatient department ≥2 |
| Diabetes mellitus | 250 | E11-E14 | Outpatient department ≥2 |
| Hyperlipidemia | 272 | E78 | Outpatient department ≥2 |
| Chronic gout | 274.0, 274.10, 274.11, 274.19, 274.81, 274.82, 274.89, 274.9 | M10, M1A | Outpatient department ≥2 |
| Chronic lung disease | 490, 491.0, 491.1, 491.20-491.22, 491.8, 491.9, 492.0, 492.8, 493.00-493.02 493.10-493.12, 493.20-493.22, 493.81, 493.82, 493.90-493.92, 494.0, 494.1, 495.8, 495.9, 496, 500, 502, 503, 504, 505, A323, A325 | J41-J44 | Discharge |
| Chronic kidney disease | 580-589 | I12, I13, N00, N01, N02, N03, N04, N05, N07, N11, N14, N17, N18, N19, Q61 | Outpatient department ≥2 |
| Chronic liver disease | 570, 571, 572 | B150, B160, B162, B190, K704, K72, K766, I85 | Outpatient department ≥2 |
| Malignancy | 140.0-208.9 | C | Outpatient department ≥2 |
| Intracranial hemorrhage (ICH) | 430, 431, 432, 852, 853 | I60, I61, I62 | Discharge |
| Gastrointestinal bleeding (GIB) | 456.0, 456.2, 455.2, 455.5, 455.8, 530.7, 530.82, 531.0-531.6, 532.0-532.6, 533.0-533.6, 534.0-534.6, 535.0-535.6 537.83, 562.02, 562.03, 562.12 562.13 568.81, 569.3, 569.85, 578.0, 578.1, 578.9 | K22.6, K25.0, K25.2, K25.4, K25.6, K26.0, K26.2, K26.4, K26.6, K27.0, K27.2, K27.4, K27.6, K28.0, K28.2, K28.4, K28.6, K29.0, K62.5, K92.0, K92.1, K92.2 | Discharge |
| Other critical site bleeding | 423,0, 459.0, 568.81, 593.81, 599.7, 623.8, 626.32, 626.6, 719.1, 784.7, 784.8, 786.3 | D62, J942, H113, H356, H431, N02, N95, R04, R31, R58 | Discharge |

**Table S2**

International Classification of Disease (9^th^ and 10^th^ edition) Clinical Modification (ICD 9-CM and ICD 10-CM) codes used to define the major adverse limb outcome in the study cohort

| Disease | ICD-9 Codes | ICD-10 Codes | Diagnosis definition |
| --- | --- | --- | --- |
| Revascularization  (Procedural codes) | 38.08 (incision of vessel, lower limb arteries) | 041,045,047,049,04B,04C,04H,  04J,04L,04N,04P,04Q,04R,04S,  04U,04V,04W  Location:(C,D,E,F,H,J,K,L,  M,N,P,Q,R,S,T,U,V,W,Y) | Discharge |
|  | 38.18 (endarterectomy, lower limb arteries) |  |  |
|  | 38.38 (resection of vessel with anastomosis, lower limb arteries) |  |  |
|  | 38.48 (resection of vessel with replacement, lower limb arteries) |  |  |
|  | 38.68 (other excision of vessel, lower limb arteries) |  |  |
|  | 38.88 (other surgical occlusion of vessel, lower limb arteries) |  |  |
|  | 39.50 (angioplasty or atherectomy of non-coronary vessel) |  |  |
|  | 39.7 (Endovascular repair of vessel) |  |  |
|  | 39.90 (insertion of non-coronary artery stent) |  |  |
|  | 39.25 (aorta-iliac-femoral bypass) |  |  |
|  | 39.26 (other intra-abdominal vascular shunt or bypass) |  |  |
|  | 39.29 (other(peripheral) vascular shunt or bypass) |  |  |
| Amputation | - 1. (amputation of lower limb) | 0Y67, 0Y68, 0Y6C, 0Y6D, 0Y6F, 0Y6G, 0Y6H, 0Y6J, 0Y6M, 0Y6N, 0Y6P, 0Y6Q, 0Y6R, 0Y6S, 0Y6T, 0Y6U, 0Y6V, 0Y6W, 0Y6X, 0Y6Y |  |
|  | 84.10 ~ 84.15 low level amputation (amputation below knee) |  |  |
|  | 84.16 ~ 84.19 high level amputation (knee disarticulation or above) |  |  |

**Figure S1. Cumulative incidence curves of outcomes for atrial fibrillation (AF) patients with concomitant diabetes mellitus (DM) taking oral anticoagulants before propensity score stabilized weighting (PSSW).**

Cumulative incidence curves of effectiveness outcomes including ischemic stroke/systemic embolism (IS/SE), acute myocardial infarction (AMI), and major adverse cardiovascular events (MACE) **(A)**, major adverse limb events including lower extremity revascualization procedure, lower limb amputation, and major adverse limb events (MALE) **(B),** and safety outcomes including intracranaial hemorrhage (ICH), major gastrointestinal bleeding, and all major bleeding **(C)** for AF patients with concomiant DM taking oral anticoagulants before PSSW are presented. NOAC use was associated with a lower risk of MACE, MALE, and all major bleeding compared to warfarin among AF patients with concomitant DM.

Abbreviations: AF = atrial fibrillation; AMI = acute myocardial infarction; DM = diabetes mellitus; ICH = intracranial hemorrhage; IS/SE = ischemic stroke/systemic embolism; MACE = major adverse cardiovascular events; MALE = major adverse limb events; NOAC = non-vitamin K antagonist oral anticoagulants; PSSW = propensity score stabilized weighting

**Figure S2. Forest plot of hazard ratio (HR) of effectiveness, major lower limb outcomes, and safety outcomes for NOACs vs. warfarin among non-valvular AF patients comorbid with DM, after multi-variate adjustment.**

Consistent with the main analysis by using PSSW, NOAC use was associated with a lower risk of MACE, MALE, and major bleeding than warfarin among non-valvular AF patients with concomitant DM, after multivariate Cox’s proportional hazards model which included the baseline covariates in Table 1 except for CHA_2_DS_2_-VASc and HAS-BLED scores.

Abbreviations: AF = atrial fibrillation; AMI = acute myocardial infarction; CI = confidential interval; GI = gastrointestinal; HR = hazard ratio; ICH = intracranial hemorrhage; IS/SE = ischemic stroke/systemic embolism; MACE = major adverse cardiovascular events; MALE = major adverse limb events; NOAC = non-vitamin K antagonist oral anticoagulants; PSSW = propensity score stabilized weighting
