# Supplementary figures and images for: Effectiveness, safety, and major adverse limb events in atrial fibrillation patients with concomitant diabetes mellitus treated with non-vitamin K antagonist oral anticoagulants
Source: Cardiovasc Diabetol. 2020 May 13;19:63. doi: 10.1186/s12933-020-01043-2 (PMC7222472; doi:10.1186/s12933-020-01043-2)

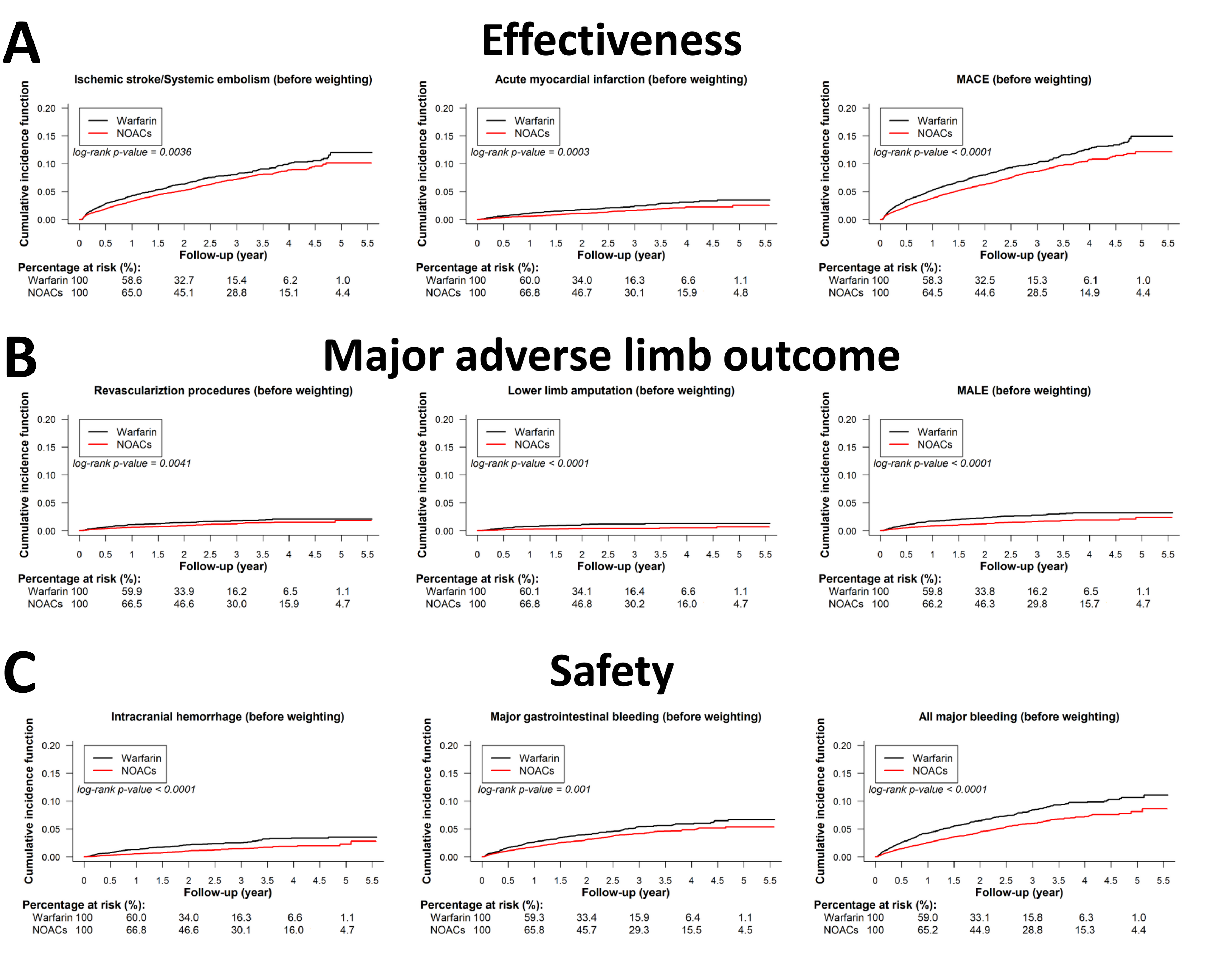

Supplement: Supplementary file 2 — Additional file 2: Figure S1. Cumulative incidence curves of outcomes for atrial fibrillation (AF) patients with concomitant diabetes mellitus (DM) taking oral anticoagulants before propensity score stabilized weighting (PSSW). [file 12933_2020_1043_MOESM2_ESM.tiff]

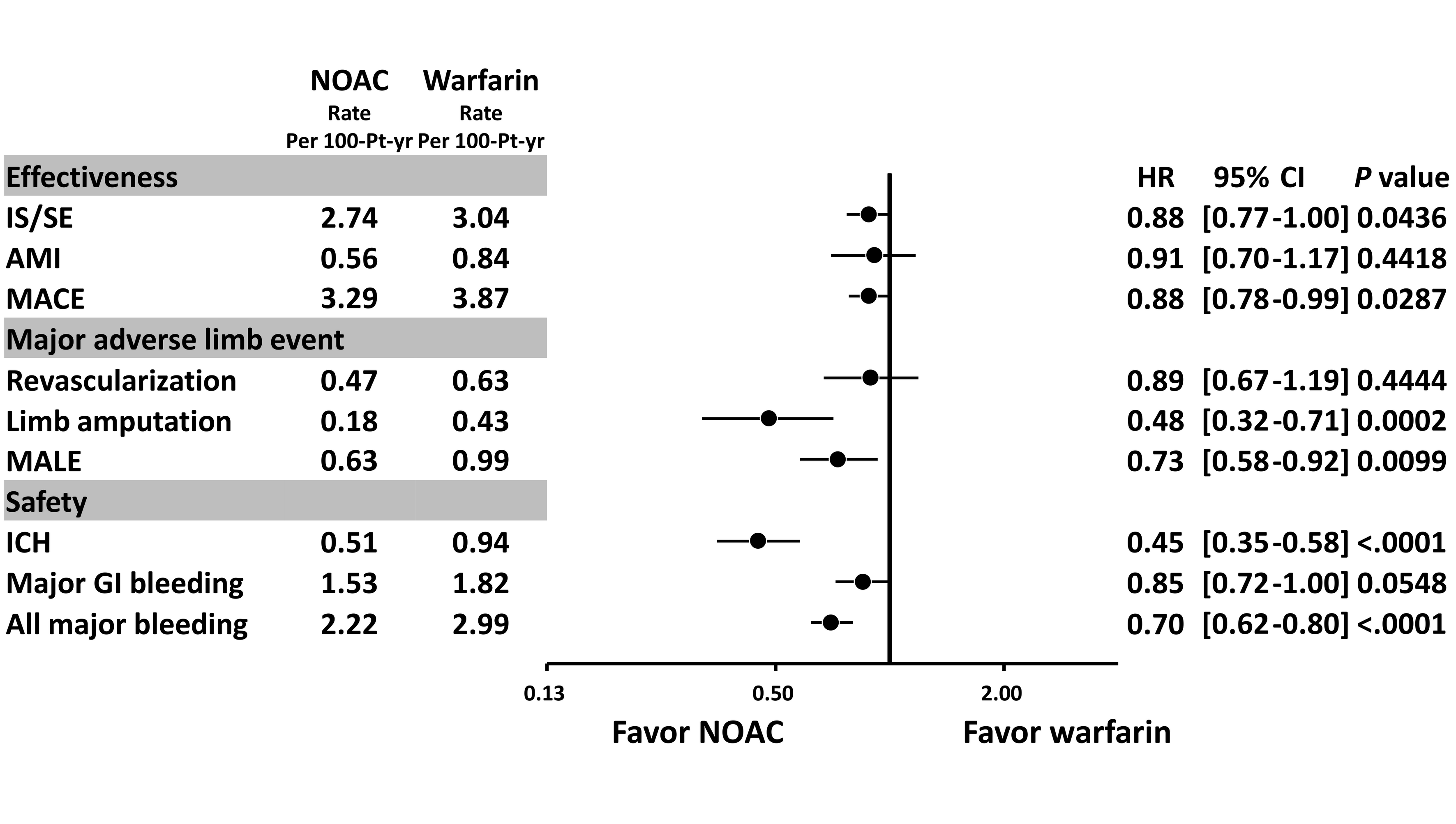

Supplement: Supplementary file 3 — Additional file 3: Figure S2. Forest plot of hazard ratio (HR) of effectiveness, major lower limb outcomes, and safety outcomes for NOACs vs. warfarin among non-valvular AF patients comorbid with DM, after multi-variate adjustment. [file 12933_2020_1043_MOESM3_ESM.tiff]
